# Medical Education

**Published:** 1857-09

**Authors:** 


### EDITORIAL.

*Medical Education.*

We are gratified to see that this subject is, at the present time, receiving much attention from our brethren of the medical press. It has for some months past occupied a prominent place in the columns of the *North American Medico-Chirurgical Review*; the *Medical Gazette*, of New York; the *Stethescope*, of Richmond, Va.; the *Medical and Surgical Reporter*, of N. J.; both the New Orleans Medical Journals; the *Southern Journal of Medical and Physical Sciences*; the *Nashville Journal*; the *Medical Observer*, of Cincinnati; the *Medical Independent*, of Detroit, and some others. Nearly all the writers on this subject advocate three leading ideas, viz: 1st, A more extended and reasonable degree of preliminary education for the student before he enters upon the study of medicine. 2d, Longer College terms, so as to admit of more complete instruction in the various departments. 3d, The adoption of more systematic and extensive arrangements for clinical instruction.

The views of the editor of this journal, in relation to all these topics, have been before the profession several years, and may be found fully expressed in the last chapter of the small volume entitled "A History of Medical Education and Institutions in the United States," and also in the more recent work entitled "A History of the American Medical Association."

We have, in both of the above named works, in the columns of this journal, and on all other suitable occasions, advocated each of the before named propositions. The first, we regard as the primary step, absolutely essential, as the only basis for real advancement in all other respects. But this matter of preliminary education belongs emphatically to the profession at large, and not to the schools. The proper standard of preliminary education must be exacted as the first initiatory step when the young man applies to the practitioner for admission into his office as a student of medicine. This was the position assumed by the American Medical Association on the first completion of its organization in 1847; and, two years after, it went further, and distinctly recommended that each County or local Society adopt such regulations as will prohibit its members from receiving any students into their offices until they exhibit ample proof that they possess the requisite knowledge of the ordinary branches of science. Some of the recent writers on this subject have recommended that the National Association refuse to receive delegates from all such Medical Colleges as do not fully comply with the regulations it may adopt.

When this is done, we shall move that the Association also refuse to receive delegates from all such State, County and local Societies, as do not effectually prohibit their members from receiving as medical students, young men whose preliminary education is grossly defective.

In regard to the second proposition, that of lengthening the College term, we are more fully satisfied with each additional year's experience, that no more extension of the College terms to five or six months will accomplish any of the important objects desired. Our brethren who desire to see the present system of College instruction improved, must remember that the entire want of method, or in other words, the custom of lecturing on all the branches of medicine to the same classes on the same day, is quite as great an evil as the want of time in a four month's term. To make any substantial improvement we must extend the present College term to a Collegiate year of nine or ten months, and divide it into two or three subdivisions or minor terms, so that a limited number of branches or departments of Medical Science may be taught in each. By judiciously grouping the several subjects, each minor division of the Collegiate year would present such topics as are suited to the student at each successive year of his advancement.

He would thus be able to pursue the several branches in their natural order and relation to each other, attending such division of the general College term each year as corresponded with his progress in study. This would reduce the study of medicine to a methodical and well digested system; and we should no longer witness the absurd practice of lecturing to young men on the details of practical medicine and surgery before they had studied long enough to become familiar with the bones of the skeleton or the technical language of the Materia Medica. We have advocated this change for several years, not merely in the two published volumes mentioned above, but in the columns of this journal, and also in the lecture room of the College with which we are connected; and we were very much gratified to see it taken up and distinctly advocated in the pages of so influential a journal as the *North American Medico-Chirurgical Review*. In relation to the third proposition, calling for a more systematic and extensive system of clinical instruction, we think there are indications of genuine progress. These indications are not only visible in the action of the several State and National organizations, and the discussions of the medical journals, but also in the action of such Colleges as have heretofore omitted to provide any facilities for this kind of instruction. Even the Medical Department of the Michigan University has been compelled to change its tactics, and actually get up some *show* of clinical instruction, by inviting its students to a summer course in the hospital at Detroit. We say *inviting*, because it has not yet come up to the standard adopted several years since by the best schools in the Union, and made attendance on a course of Hospital Clinical Instruction one of the requisitions for graduation. We are glad, however, to see even the smallest indications of progress in this important department of medical education. It is now seven years since the Rush Medical College with which we are connected, adopted a rule making attendance upon at least one course of Hospital Clinical Instruction a requisition for graduation. The Mercy Hospital, to the wards of which we have unrestrained access, always contains a number of patients, both medical and surgical, greater than can be profitably used for clinical purposes.

The regular course of instruction is commenced on the first Monday in October in each year, and an hour is devoted to the class in the wards, every morning (Sundays excepted) from that time to the 1st of March. From the last named date to the 1st of July clinical instruction is continued three mornings in the week: thus giving all those who take the hospital ticket the privilege of clinical instruction nine months of the year. We cheerfully invite, to this department of our College facilities, the closest scrutiny of the profession.

